# Supplementary material for: Genomic Insights into the Symbiotic and Plant Growth-Promoting Traits of “Candidatus Phyllobacterium onerii” sp. nov. Isolated from Endemic Astragalus flavescens
Source: Microorganisms. 2024 Feb 6;12(2):336. doi: 10.3390/microorganisms12020336 (PMC10891626; doi:10.3390/microorganisms12020336)
Supplement: Supplementary file 1 [file microorganisms-12-00336-s001.zip › microorganisms-2816636-SI.pdf]

Supplementary Table S1. Primers and PCR conditions

**Target site**	**Primer**	**Annealing °C**	**Reference**
**16S rRNA**	27F: (5′AGAGTTTGATCCTGGCTCAG 3′)		Koskey
	1492R: (5′ GGTTACCTTGTTACGACTT 3′)	58	et al., 2018
recA	*recA*F: (5′ATC GAG CGG TCG TTC GGC AAG GG 3′)		Gaunt
	*recA*R: (5′ TTG CGC AGC GCC TGG CTC AT 3′)	56	et al., 2001

Supplementary Table S2. Several genes involved in carbohydrate metabolism

	**Subcategory**	**Subsystem**	**Role**
1	Monosaccharides	Xylose utilization	Xylulose kinase (EC 2.7.1.17)
2	Monosaccharides	Xylose utilization	Putative xylulose kinase (EC 2.7.1.17)
3	Monosaccharides	Xylose utilization	Xylose-responsive transcription regulator, ROK family
4	Monosaccharides	Xylose utilization	Xylose isomerase (EC 5.3.1.5)
5	Monosaccharides	D-gluconate and ketogluconates metabolism	Gluconate dehydratase (EC 4.2.1.39)
6	Monosaccharides	D-gluconate and ketogluconates metabolism	L-idonate 5-dehydrogenase (EC 1.1.1.264)
7	Monosaccharides	D-gluconate and ketogluconates metabolism	2,5-diketo-D-gluconic acid reductase B (EC 1.1.1.274)
8	Monosaccharides	D-gluconate and ketogluconates metabolism	5-keto-D-gluconate 5-reductase (EC 1.1.1.69)
9	Monosaccharides	D-gluconate and ketogluconates metabolism	Gluconokinase (EC 2.7.1.12)
10	Monosaccharides	D-gluconate and ketogluconates metabolism	Glucose dehydrogenase, PQQ-dependent (EC 1.1.5.2)
11	Monosaccharides	D-gluconate and ketogluconates metabolism	6-phosphogluconate dehydrogenase, decarboxylating (EC 1.1.1.44)
12	Monosaccharides	Deoxyribose and Deoxynucleoside Catabolism	Purine nucleoside phosphorylase (EC 2.4.2.1)
13	Monosaccharides	Deoxyribose and Deoxynucleoside Catabolism	Predicted nucleoside ABC transporter, permease 1 component
14	Monosaccharides	Deoxyribose and Deoxynucleoside Catabolism	Deoxyribose-phosphate aldolase (EC 4.1.2.4)
15	Monosaccharides	Deoxyribose and Deoxynucleoside Catabolism	Thymidine phosphorylase (EC 2.4.2.4)

16	Monosaccharides	Deoxyribose and Deoxynucleoside Catabolism	Phosphopentomutase (EC 5.4.2.7)
17	Monosaccharides	Deoxyribose and Deoxynucleoside Catabolism	Ribokinase (EC 2.7.1.15)
18	Monosaccharides	Deoxyribose and Deoxynucleoside Catabolism	Predicted nucleoside ABC transporter, ATP-binding component
19	Monosaccharides	D-ribose utilization	Ribokinase (EC 2.7.1.15)
20	Monosaccharides	D-ribose utilization	Predicted nucleoside ABC transporter, ATP-binding component
21	Monosaccharides	D-ribose utilization	Ribose/xylose/arabinose/galactoside ABC-type transport systems, permease component 1
22	Monosaccharides	D-ribose utilization	Predicted nucleoside ABC transporter, permease 1 component
23	Monosaccharides	Fructose utilization	Phosphoenolpyruvate-protein phosphotransferase of PTS system (EC 2.7.3.9)
24	Monosaccharides	Fructose utilization	Transcriptional regulator FrcR for fructose utilization, ROK family
25	Monosaccharides	Fructose utilization	Fructokinase (EC 2.7.1.4)
26	Monosaccharides	Fructose utilization	Fructose ABC transporter, ATP-binding component FrcA
27	Monosaccharides	Fructose utilization	Fructose ABC transporter, permease component FrcC
28	Monosaccharides	Fructose utilization	Fructose ABC transporter, substrate-binding component FrcB
29	Monosaccharides	Fructose utilization	Phosphotransferase system, phosphocarrier protein HPr
30	Monosaccharides	Fructose utilization	Transaldolase (EC 2.2.1.2)
31	Monosaccharides	D-galactonate catabolism	2-dehydro-3-deoxyphosphogalactonate aldolase (EC 4.1.2.21)
32	Monosaccharides	D-galactonate catabolism	2-dehydro-3-deoxygalactonokinase (EC 2.7.1.58)
33	Monosaccharides	D-Galacturonate and D-Glucuronate Utilization	5-dehydro-4-deoxyglucarate dehydratase (EC 4.2.1.41)
34	Monosaccharides	D-Galacturonate and D-Glucuronate Utilization	Pectin degradation protein KdgF
35	Monosaccharides	D-Galacturonate and D-Glucuronate Utilization	4-deoxy-L-threo-5-hexosulose-uronate ketol-isomerase (EC 5.3.1.17)
36	Monosaccharides	D-Galacturonate and D-Glucuronate Utilization	2-deoxy-D-gluconate 3-dehydrogenase (EC 1.1.1.125)
37	Monosaccharides	D-Galacturonate and D-Glucuronate Utilization	Mannonate dehydratase (EC 4.2.1.8)
38	Monosaccharides	D-Galacturonate and D-Glucuronate Utilization	Uronate isomerase (EC 5.3.1.12)
39	Monosaccharides	D-Galacturonate and D-Glucuronate Utilization	2-dehydro-3-deoxy-D-gluconate 5-dehydrogenase (EC 1.1.1.127)
40	Monosaccharides	D-Galacturonate and D-Glucuronate Utilization	2-dehydro-3-deoxyphosphogluconate aldolase (EC 4.1.2.14)
41	Monosaccharides	D-Galacturonate and D-Glucuronate Utilization	D-mannonate oxidoreductase (EC 1.1.1.57)
42	Monosaccharides	L-fucose utilization temp	L-fuco-beta-pyranose dehydrogenase (EC 1.1.1.122)
43	Monosaccharides	L-fucose utilization temp	L-fuconate dehydratase (EC 4.2.1.68)
44	Monosaccharides	L-fucose utilization temp	2-keto-3-deoxy-L-fuconate dehydrogenase
45	Monosaccharides	L-fucose utilization temp	L-fuconolactone hydrolase
46	Monosaccharides	L-fucose utilization temp	2,4-diketo-3-deoxy-L-fuconate hydrolase

47	Aminosugars	Chitin and N-acetylglucosamine utilization	N-acetylglucosamine kinase of eukaryotic type (EC 2.7.1.59)
48	Aminosugars	Chitin and N-acetylglucosamine utilization	Predicted transcriptional regulator of N-Acetylglucosamine utilization, GntR family
49	Aminosugars	Chitin and N-acetylglucosamine utilization	N-acetylglucosamine-6-phosphate deacetylase (EC 3.5.1.25)
50	Aminosugars	Chitin and N-acetylglucosamine utilization	N-Acetyl-D-glucosamine ABC transport system ATP-binding protein
51	Aminosugars	Chitin and N-acetylglucosamine utilization	Glucosamine-6-phosphate deaminase [isomerizing], alternative (EC 3.5.99.6)
52	Aminosugars	Chitin and N-acetylglucosamine utilization	hypothetical oxidoreductase related to N-acetylglucosamine utilization
53	Di- and oligosaccharides	Maltose and Maltodextrin Utilization	Maltose/maltodextrin transport ATP-binding protein MalK (EC 3.6.3.19)
54	Di- and oligosaccharides	Maltose and Maltodextrin Utilization	Maltose/maltodextrin ABC transporter, permease protein MalG
55	Di- and oligosaccharides	Maltose and Maltodextrin Utilization	Malto-oligosyltrehalose synthase (EC 5.4.99.15)
56	Di- and oligosaccharides	Maltose and Maltodextrin Utilization	Alpha-amylase (EC 3.2.1.1)
57	Di- and oligosaccharides	Maltose and Maltodextrin Utilization	4-alpha-glucanotransferase (amylomaltase) (EC 2.4.1.25)
58	Di- and oligosaccharides	Maltose and Maltodextrin Utilization	Aldose 1-epimerase (EC 5.1.3.3)
59	Di- and oligosaccharides	Trehalose Biosynthesis	Trehalose synthase (EC 5.4.99.16)
60	Di- and oligosaccharides	Trehalose Biosynthesis	Alpha-amylase (EC 3.2.1.1)
61	Di- and oligosaccharides	Trehalose Biosynthesis	Malto-oligosyltrehalose synthase (EC 5.4.99.15)
62	Di- and oligosaccharides	Trehalose Biosynthesis	Malto-oligosyltrehalose trehalohydrolase (EC 3.2.1.141)
63	Di- and oligosaccharides	Trehalose Biosynthesis	Trehalose-6-phosphate phosphatase (EC 3.1.3.12)
64	Di- and oligosaccharides	Trehalose Biosynthesis	1,4-alpha-glucan (glycogen) branching enzyme, GH-13-type (EC 2.4.1.18)
65	Di- and oligosaccharides	Trehalose Biosynthesis	Alpha,alpha-trehalose-phosphate synthase [UDP-forming] (EC 2.4.1.15)
66	Carbohydrates - no subcategory	VC0266	Hypothetical protein VC0266 (sugar utilization related?)
67	Organic acids	Lactate utilization	L-lactate permease
68	Organic acids	Lactate utilization	L-lactate dehydrogenase (EC 1.1.2.3)
69	CO2 fixation	CO2 uptake, carboxysome	Ribulose bisphosphate carboxylase small chain (EC 4.1.1.39)
70	CO2 fixation	CO2 uptake, carboxysome	RuBisCO operon transcriptional regulator CbbR
71	CO2 fixation	CO2 uptake, carboxysome	Ribulose bisphosphate carboxylase large chain (EC 4.1.1.39)
72	CO2 fixation	Photorespiration (oxidative C2 cycle)	D-Lactate dehydrogenase, cytochrome c-dependent (EC 1.1.2.4)
73	CO2 fixation	Photorespiration (oxidative C2 cycle)	Glyoxylate carboligase (EC 4.1.1.47)
74	CO2 fixation	Photorespiration (oxidative C2 cycle)	Phosphoglycolate phosphatase (EC 3.1.3.18)
75	CO2 fixation	Photorespiration (oxidative C2 cycle)	Ribulose bisphosphate carboxylase large chain (EC 4.1.1.39)
76	CO2 fixation	Photorespiration (oxidative C2 cycle)	2-hydroxy-3-oxopropionate reductase (EC 1.1.1.60)
77	CO2 fixation	Photorespiration (oxidative C2 cycle)	Glycine cleavage system H protein

78	CO2 fixation	Photorespiration (oxidative C2 cycle)	Glycine dehydrogenase [decarboxylating] (glycine cleavage system P protein) (EC 1.4.4.2)
79	CO2 fixation	Photorespiration (oxidative C2 cycle)	Ribulose bisphosphate carboxylase small chain (EC 4.1.1.39)
80	CO2 fixation	Photorespiration (oxidative C2 cycle)	Glycolate dehydrogenase (EC 1.1.99.14), subunit GlcD
81	CO2 fixation	Photorespiration (oxidative C2 cycle)	Serine hydroxymethyltransferase (EC 2.1.2.1)
82	CO2 fixation	Photorespiration (oxidative C2 cycle)	Glycolate dehydrogenase (EC 1.1.99.14), FAD-binding subunit GlcE
83	CO2 fixation	Photorespiration (oxidative C2 cycle)	Malate synthase G (EC 2.3.3.9)
84	CO2 fixation	Photorespiration (oxidative C2 cycle)	Glycolate dehydrogenase (EC 1.1.99.14), iron-sulfur subunit GlcF
85	CO2 fixation	Photorespiration (oxidative C2 cycle)	Hydroxypyruvate reductase (EC 1.1.1.81)
86	CO2 fixation	Photorespiration (oxidative C2 cycle)	Aminomethyltransferase (glycine cleavage system T protein) (EC 2.1.2.10)
87	One-carbon Metabolism	One-carbon metabolism by tetrahydropterines	Methylenetetrahydrofolate dehydrogenase (NADP+) (EC 1.5.1.5)
88	One-carbon Metabolism	One-carbon metabolism by tetrahydropterines	5-formyltetrahydrofolate cyclo-ligase (EC 6.3.3.2)
89	One-carbon Metabolism	One-carbon metabolism by tetrahydropterines	Methenyltetrahydrofolate cyclohydrolase (EC 3.5.4.9)
90	One-carbon Metabolism	One-carbon metabolism by tetrahydropterines	Formate--tetrahydrofolate ligase (EC 6.3.4.3)
91	One-carbon Metabolism	One-carbon metabolism by tetrahydropterines	Methylene tetrahydromethanopterin dehydrogenase (EC 1.5.99.9)
92	One-carbon Metabolism	One-carbon metabolism by tetrahydropterines	5,10-methylenetetrahydrofolate reductase (EC 1.5.1.20)
93	One-carbon Metabolism	One-carbon metabolism by tetrahydropterines	Formyltetrahydrofolate deformylase (EC 3.5.1.10)
94	One-carbon Metabolism	One-carbon metabolism by tetrahydropterines	N(5),N(10)-methenyltetrahydromethanopterin cyclohydrolase (EC 3.5.4.27)
95	One-carbon Metabolism	Methanogenesis	Formylmethanofuran--tetrahydromethanopterin N-formyltransferase (EC 2.3.1.101)
96	One-carbon Metabolism	Methanogenesis	Formylmethanofuran dehydrogenase subunit A (EC 1.2.99.5)
97	One-carbon Metabolism	Methanogenesis	N(5),N(10)-methenyltetrahydromethanopterin cyclohydrolase (EC 3.5.4.27)
98	Fermentation	Butanol Biosynthesis	3-hydroxybutyryl-CoA dehydrogenase (EC 1.1.1.157)
99	Fermentation	Butanol Biosynthesis	Enoyl-CoA hydratase (EC 4.2.1.17)
100	Fermentation	Butanol Biosynthesis	Alcohol dehydrogenase (EC 1.1.1.1)
101	Fermentation	Butanol Biosynthesis	Acetyl-CoA acetyltransferase (EC 2.3.1.9)
102	Fermentation	Acetyl-CoA fermentation to Butyrate	Electron transfer flavoprotein, alpha subunit
103	Fermentation	Acetyl-CoA fermentation to Butyrate	3-hydroxyacyl-CoA dehydrogenase (EC 1.1.1.35)
104	Fermentation	Acetyl-CoA fermentation to Butyrate	3-hydroxybutyryl-CoA dehydrogenase (EC 1.1.1.157)
105	Fermentation	Acetyl-CoA fermentation to Butyrate	Electron transfer flavoprotein-ubiquinone oxidoreductase (EC 1.5.5.1)
106	Fermentation	Acetyl-CoA fermentation to Butyrate	Acetoacetyl-CoA reductase (EC 1.1.1.36)
107	Fermentation	Acetyl-CoA fermentation to Butyrate	3-hydroxybutyryl-CoA epimerase (EC 5.1.2.3)
108	Fermentation	Acetyl-CoA fermentation to Butyrate	3-hydroxybutyrate dehydrogenase (EC 1.1.1.30)

109	Fermentation	Acetyl-CoA fermentation to Butyrate	Electron transfer flavoprotein, beta subunit
110	Fermentation	Acetyl-CoA fermentation to Butyrate	3-hydroxybutyryl-CoA dehydratase (EC 4.2.1.55)
111	Fermentation	Acetyl-CoA fermentation to Butyrate	Enoyl-CoA hydratase (EC 4.2.1.17)
112	Fermentation	Acetyl-CoA fermentation to Butyrate	Acetyl-CoA acetyltransferase (EC 2.3.1.9)
113	Fermentation	Acetolactate synthase subunits	Acetolactate synthase small subunit (EC 2.2.1.6)
114	Fermentation	Acetolactate synthase subunits	Acetolactate synthase large subunit (EC 2.2.1.6)
115	Sugar alcohols	Glycerol and Glycerol-3-phosphate Uptake and Utilization	Glycerol-3-phosphate ABC transporter, permease protein UgpA (TC 3.A.1.1.3)
116	Sugar alcohols	Glycerol and Glycerol-3-phosphate Uptake and Utilization	Glycerol-3-phosphate regulon repressor GlpR
117	Sugar alcohols	Glycerol and Glycerol-3-phosphate Uptake and Utilization	Glycerophosphoryl diester phosphodiesterase (EC 3.1.4.46)
118	Sugar alcohols	Glycerol and Glycerol-3-phosphate Uptake and Utilization	Glycerol-3-phosphate ABC transporter, permease protein UgpE (TC 3.A.1.1.3)
119	Sugar alcohols	Glycerol and Glycerol-3-phosphate Uptake and Utilization	Glycerol kinase (EC 2.7.1.30)
120	Sugar alcohols	Glycerol and Glycerol-3-phosphate Uptake and Utilization	Glycerol-3-phosphate dehydrogenase [NAD(P)+] (EC 1.1.1.94)
121	Sugar alcohols	Glycerol and Glycerol-3-phosphate Uptake and Utilization	Glycerol-3-phosphate ABC transporter, ATP-binding protein UgpC (TC 3.A.1.1.3)
122	Sugar alcohols	Glycerol and Glycerol-3-phosphate Uptake and Utilization	Glycerol-3-phosphate dehydrogenase (EC 1.1.5.3)
123	Sugar alcohols	Glycerol and Glycerol-3-phosphate Uptake and Utilization	Aerobic glycerol-3-phosphate dehydrogenase (EC 1.1.5.3)
124	Central carbohydrate metabolism	Methylglyoxal Metabolism	Aldehyde dehydrogenase B (EC 1.2.1.22)
125	Central carbohydrate metabolism	Methylglyoxal Metabolism	Aldehyde dehydrogenase (EC 1.2.1.3)
126	Central carbohydrate metabolism	Methylglyoxal Metabolism	Methylglyoxal synthase (EC 4.2.3.3)
127	Central carbohydrate metabolism	Methylglyoxal Metabolism	Lactoylglutathione lyase (EC 4.4.1.5)
128	Central carbohydrate metabolism	Methylglyoxal Metabolism	Hydroxyacylglutathione hydrolase (EC 3.1.2.6)
129	Central carbohydrate metabolism	Pyruvate metabolism I: anaplerotic reactions, PEP	Pyruvate kinase (EC 2.7.1.40)
130	Central carbohydrate metabolism	Pyruvate metabolism I: anaplerotic reactions, PEP	Phosphoenolpyruvate carboxykinase [ATP] (EC 4.1.1.49)
131	Central carbohydrate metabolism	Pyruvate metabolism I: anaplerotic reactions, PEP	Phosphoenolpyruvate synthase (EC 2.7.9.2)
132	Central carbohydrate metabolism	Pyruvate metabolism I: anaplerotic reactions, PEP	Pyruvate,phosphate dikinase (EC 2.7.9.1)
133	Central carbohydrate metabolism	Pyruvate metabolism I: anaplerotic reactions, PEP	NAD-dependent malic enzyme (EC 1.1.1.38)
134	Central carbohydrate metabolism	Pyruvate metabolism I: anaplerotic reactions, PEP	Phosphoenolpyruvate carboxylase (EC 4.1.1.31)
135	Central carbohydrate metabolism	Glyoxylate bypass	Citrate synthase (si) (EC 2.3.3.1)
136	Central carbohydrate metabolism	Glyoxylate bypass	Malate synthase G (EC 2.3.3.9)
137	Central carbohydrate metabolism	Glyoxylate bypass	Malate dehydrogenase (EC 1.1.1.37)
138	Central carbohydrate metabolism	Glyoxylate bypass	(R)-2-hydroxyacid dehydrogenase, similar to L-sulfolactate dehydrogenase (EC 1.1.1.272)
139	Central carbohydrate metabolism	Glyoxylate bypass	Aconitate hydratase (EC 4.2.1.3)

140	Central carbohydrate metabolism	Glyoxylate bypass	Isocitrate lyase (EC 4.1.3.1)
141	Central carbohydrate metabolism	Glycolate, glyoxylate interconversions	Hydroxypyruvate reductase (EC 1.1.1.81)
142	Central carbohydrate metabolism	Glycolate, glyoxylate interconversions	Glyoxylate reductase (EC 1.1.1.79)
143	Central carbohydrate metabolism	Glycolate, glyoxylate interconversions	Glycolate dehydrogenase (EC 1.1.99.14), subunit GlcD
144	Central carbohydrate metabolism	Glycolate, glyoxylate interconversions	Glycolate dehydrogenase (EC 1.1.99.14), iron-sulfur subunit GlcF
145	Central carbohydrate metabolism	Glycolate, glyoxylate interconversions	Glycolate dehydrogenase (EC 1.1.99.14), FAD-binding subunit GlcE
146	Central carbohydrate metabolism	Glycolate, glyoxylate interconversions	Phosphoglycolate phosphatase (EC 3.1.3.18)
147	Central carbohydrate metabolism	Glycolate, glyoxylate interconversions	D-Lactate dehydrogenase, cytochrome c-dependent (EC 1.1.2.4)
148	Central carbohydrate metabolism	Glycolate, glyoxylate interconversions	Glyoxylate reductase (EC 1.1.1.26)
149	Central carbohydrate metabolism	TCA Cycle	hypothetical protein that often co-occurs with aconitase
150	Central carbohydrate metabolism	TCA Cycle	Isocitrate dehydrogenase [NADP] (EC 1.1.1.42)
151	Central carbohydrate metabolism	TCA Cycle	Aconitate hydratase (EC 4.2.1.3)
152	Central carbohydrate metabolism	TCA Cycle	Dihydrolipoamide dehydrogenase of 2-oxoglutarate dehydrogenase (EC 1.8.1.4)
153	Central carbohydrate metabolism	TCA Cycle	Dihydrolipoamide dehydrogenase of pyruvate dehydrogenase complex (EC 1.8.1.4)
154	Central carbohydrate metabolism	TCA Cycle	Dihydrolipoamide succinyltransferase component (E2) of 2-oxoglutarate dehydrogenase cor
155	Central carbohydrate metabolism	TCA Cycle	Malate dehydrogenase (EC 1.1.1.37)
156	Central carbohydrate metabolism	TCA Cycle	Fumarate hydratase class II (EC 4.2.1.2)
157	Central carbohydrate metabolism	TCA Cycle	Succinyl-CoA ligase [ADP-forming] alpha chain (EC 6.2.1.5)
158	Central carbohydrate metabolism	TCA Cycle	Succinyl-CoA ligase [ADP-forming] beta chain (EC 6.2.1.5)
159	Central carbohydrate metabolism	TCA Cycle	Citrate synthase (si) (EC 2.3.3.1)
160	Central carbohydrate metabolism	TCA Cycle	2-oxoglutarate dehydrogenase E1 component (EC 1.2.4.2)
161	Central carbohydrate metabolism	Glycolysis and Gluconeogenesis	Fructose-bisphosphate aldolase class I (EC 4.1.2.13)
162	Central carbohydrate metabolism	Glycolysis and Gluconeogenesis	Pyrophosphate-dependent fructose 6-phosphate-1-kinase (EC 2.7.1.90)
163	Central carbohydrate metabolism	Glycolysis and Gluconeogenesis	Glucose-6-phosphate isomerase (EC 5.3.1.9)
164	Central carbohydrate metabolism	Glycolysis and Gluconeogenesis	Glucokinase (EC 2.7.1.2)
165	Central carbohydrate metabolism	Glycolysis and Gluconeogenesis	Phosphoglycerate kinase (EC 2.7.2.3)
166	Central carbohydrate metabolism	Glycolysis and Gluconeogenesis	Fructose-bisphosphate aldolase class II (EC 4.1.2.13)
167	Central carbohydrate metabolism	Glycolysis and Gluconeogenesis	Enolase (EC 4.2.1.11)
168	Central carbohydrate metabolism	Glycolysis and Gluconeogenesis	Pyruvate,phosphate dikinase (EC 2.7.9.1)
169	Central carbohydrate metabolism	Glycolysis and Gluconeogenesis	NAD-dependent glyceraldehyde-3-phosphate dehydrogenase (EC 1.2.1.12)
170	Central carbohydrate metabolism	Glycolysis and Gluconeogenesis	Fructose-1,6-bisphosphatase, GlpX type (EC 3.1.3.11)

171	Central carbohydrate metabolism	Glycolysis and Gluconeogenesis	Fructose-1,6-bisphosphatase, type I (EC 3.1.3.11)
172	Central carbohydrate metabolism	Glycolysis and Gluconeogenesis	Pyruvate kinase (EC 2.7.1.40)
173	Central carbohydrate metabolism	Glycolysis and Gluconeogenesis	Triosephosphate isomerase (EC 5.3.1.1)
174	Central carbohydrate metabolism	Glycolysis and Gluconeogenesis	Phosphoenolpyruvate synthase (EC 2.7.9.2)
175	Central carbohydrate metabolism	Pentose phosphate pathway	6-phosphogluconolactonase (EC 3.1.1.31), eukaryotic type
176	Central carbohydrate metabolism	Pentose phosphate pathway	Glucose-6-phosphate 1-dehydrogenase (EC 1.1.1.49)
177	Central carbohydrate metabolism	Pentose phosphate pathway	Ribulose-phosphate 3-epimerase (EC 5.1.3.1)
178	Central carbohydrate metabolism	Pentose phosphate pathway	Ribose-phosphate pyrophosphokinase (EC 2.7.6.1)
179	Central carbohydrate metabolism	Pentose phosphate pathway	Transketolase (EC 2.2.1.1)
180	Central carbohydrate metabolism	Pentose phosphate pathway	6-phosphogluconolactonase (EC 3.1.1.31)
181	Central carbohydrate metabolism	Pentose phosphate pathway	Transaldolase (EC 2.2.1.2)
182	Central carbohydrate metabolism	Pentose phosphate pathway	6-phosphogluconate dehydrogenase, decarboxylating (EC 1.1.1.44)
183	Central carbohydrate metabolism	Pyruvate Alanine Serine Interconversions	D-amino acid dehydrogenase small subunit (EC 1.4.99.1)
184	Central carbohydrate metabolism	Pyruvate Alanine Serine Interconversions	Omega-amino acid--pyruvate aminotransferase (EC 2.6.1.18)
185	Central carbohydrate metabolism	Pyruvate Alanine Serine Interconversions	Branched-chain amino acid aminotransferase (EC 2.6.1.42)
186	Central carbohydrate metabolism	Pyruvate Alanine Serine Interconversions	Alanine dehydrogenase (EC 1.4.1.1)
187	Central carbohydrate metabolism	Pyruvate Alanine Serine Interconversions	L-serine dehydratase, beta subunit (EC 4.3.1.17)
188	Central carbohydrate metabolism	Pyruvate Alanine Serine Interconversions	L-serine dehydratase, alpha subunit (EC 4.3.1.17)
189	Central carbohydrate metabolism	Pyruvate Alanine Serine Interconversions	Alanine racemase (EC 5.1.1.1)
190	Central carbohydrate metabolism	Pyruvate Alanine Serine Interconversions	D-alanine aminotransferase (EC 2.6.1.21)
191	Central carbohydrate metabolism	Dihydroxyacetone kinases	Phosphoenolpyruvate-dihydroxyacetone phosphotransferase (EC 2.7.1.121), subunit DhaM
192	Central carbohydrate metabolism	Dihydroxyacetone kinases	Phosphoenolpyruvate-dihydroxyacetone phosphotransferase (EC 2.7.1.121), ADP-binding s[illegible]
193	Central carbohydrate metabolism	Dihydroxyacetone kinases	DHA-specific phosphocarrier protein HPr
194	Central carbohydrate metabolism	Dihydroxyacetone kinases	Phosphoenolpyruvate-dihydroxyacetone phosphotransferase (EC 2.7.1.121), dihydroxyacet[illegible]
195	Central carbohydrate metabolism	Dihydroxyacetone kinases	Dihydroxyacetone kinase, ATP-dependent (EC 2.7.1.29)
196	Central carbohydrate metabolism	Dihydroxyacetone kinases	DHA-specific IIA component
197	Central carbohydrate metabolism	Dihydroxyacetone kinases	Hypothetical protein in cluster with dihydroxyacetone kinase in Rhizobia
198	Central carbohydrate metabolism	Dehydrogenase complexes	Branched-chain alpha-keto acid dehydrogenase, E1 component, alpha subunit (EC 1.2.4.4)
199	Central carbohydrate metabolism	Dehydrogenase complexes	Dihydrolipoamide acyltransferase component of branched-chain alpha-keto acid dehydroge[illegible]
200	Central carbohydrate metabolism	Dehydrogenase complexes	Pyruvate dehydrogenase E1 component beta subunit (EC 1.2.4.1)
201	Central carbohydrate metabolism	Dehydrogenase complexes	Cytosol aminopeptidase PepA (EC 3.4.11.1)

202	Central carbohydrate metabolism	Dehydrogenase complexes	2-oxoglutarate dehydrogenase E1 component (EC 1.2.4.2)
203	Central carbohydrate metabolism	Dehydrogenase complexes	Pyruvate dehydrogenase E1 component alpha subunit (EC 1.2.4.1)
204	Central carbohydrate metabolism	Dehydrogenase complexes	Branched-chain alpha-keto acid dehydrogenase, E1 component, beta subunit (EC 1.2.4.4)
205	Central carbohydrate metabolism	Dehydrogenase complexes	Dihydrolipoamide dehydrogenase of branched-chain alpha-keto acid dehydrogenase (EC 1.[illegible]
206	Central carbohydrate metabolism	Dehydrogenase complexes	Dihydrolipoamide acetyltransferase component of pyruvate dehydrogenase complex (EC 2.[illegible]
207	Central carbohydrate metabolism	Dehydrogenase complexes	Dihydrolipoamide succinyltransferase component (E2) of 2-oxoglutarate dehydrogenase co[illegible]
208	Central carbohydrate metabolism	Dehydrogenase complexes	Dihydrolipoamide dehydrogenase of 2-oxoglutarate dehydrogenase (EC 1.8.1.4)
209	Central carbohydrate metabolism	Pyruvate metabolism II: acetyl-CoA, acetogenesis from pyruvate	Pyruvate dehydrogenase E1 component beta subunit (EC 1.2.4.1)
210	Central carbohydrate metabolism	Pyruvate metabolism II: acetyl-CoA, acetogenesis from pyruvate	Pyruvate dehydrogenase E1 component alpha subunit (EC 1.2.4.1)
211	Central carbohydrate metabolism	Pyruvate metabolism II: acetyl-CoA, acetogenesis from pyruvate	Dihydrolipoamide dehydrogenase of pyruvate dehydrogenase complex (EC 1.8.1.4)
212	Central carbohydrate metabolism	Pyruvate metabolism II: acetyl-CoA, acetogenesis from pyruvate	Dihydrolipoamide acetyltransferase component of pyruvate dehydrogenase complex (EC 2.[illegible]
213	Central carbohydrate metabolism	Pyruvate metabolism II: acetyl-CoA, acetogenesis from pyruvate	Aldehyde dehydrogenase (EC 1.2.1.3)
214	Polysaccharides	Glycogen metabolism	1,4-alpha-glucan (glycogen) branching enzyme, GH-13-type (EC 2.4.1.18)
215	Polysaccharides	Glycogen metabolism	4-alpha-glucanotransferase (amylomaltase) (EC 2.4.1.25)

Supplementary Table S3. Several genes involved in fatty acid metabolism

	Subcategory	Subsystem	Role and EC numbers
1	Fatty Acids, Lipids, and Isoprenoids - no subcategory	Polyhydroxybutyrate metabolism	Acetyl-CoA acetyltransferase (EC 2.3.1.9)
2	Fatty Acids, Lipids, and Isoprenoids - no subcategory	Polyhydroxybutyrate metabolism	3-hydroxybutyryl-CoA dehydratase (EC 4.2.1.55)
3	Fatty Acids, Lipids, and Isoprenoids - no subcategory	Polyhydroxybutyrate metabolism	Enoyl-CoA hydratase (EC 4.2.1.17)
4	Fatty Acids, Lipids, and Isoprenoids - no subcategory	Polyhydroxybutyrate metabolism	Polyhydroxyalkanoic acid synthase
5	Fatty Acids, Lipids, and Isoprenoids - no subcategory	Polyhydroxybutyrate metabolism	3-hydroxybutyryl-CoA epimerase (EC 5.1.2.3)
6	Fatty Acids, Lipids, and Isoprenoids - no subcategory	Polyhydroxybutyrate metabolism	Acetoacetyl-CoA reductase (EC 1.1.1.36)
7	Fatty Acids, Lipids, and Isoprenoids - no subcategory	Polyhydroxybutyrate metabolism	3-hydroxybutyryl-CoA dehydrogenase (EC 1.1.1.157)
8	Fatty Acids, Lipids, and Isoprenoids - no subcategory	Polyhydroxybutyrate metabolism	3-hydroxyacyl-CoA dehydrogenase (EC 1.1.1.35)
9	Fatty Acids, Lipids, and Isoprenoids - no subcategory	Polyhydroxybutyrate metabolism	3-ketoacyl-CoA thiolase (EC 2.3.1.16)
10	Isoprenoids	Isoprenoid Biosynthesis: Interconversions	(2E,6E)-farnesyl diphosphate synthase (EC 2.5.1.10)
11	Isoprenoids	Isoprenoinds for Quinones	(2E,6E)-farnesyl diphosphate synthase (EC 2.5.1.10)
12	Isoprenoids	Isoprenoinds for Quinones	Undecaprenyl diphosphate synthase (EC 2.5.1.31)
13	Isoprenoids	Isoprenoinds for Quinones	Decaprenyl diphosphate synthase (EC 2.5.1.91)
14	Isoprenoids	Polyprenyl Diphosphate Biosynthesis	(2E,6E)-farnesyl diphosphate synthase (EC 2.5.1.10)
15	Isoprenoids	Polyprenyl Diphosphate Biosynthesis	Undecaprenyl diphosphate synthase (EC 2.5.1.31)
16	Triacylglycerols	Triacylglycerol metabolism	Lysophospholipase L2 (EC 3.1.1.5)
17	Triacylglycerols	Triacylglycerol metabolism	Monoglyceride lipase (EC 3.1.1.23)
18	Triacylglycerols	Triacylglycerol metabolism	Lysophospholipase (EC 3.1.1.5)
19	Fatty acids	Fatty acid metabolism cluster	Enoyl-CoA hydratase (EC 4.2.1.17)
20	Fatty acids	Fatty acid metabolism cluster	3-hydroxyacyl-CoA dehydrogenase (EC 1.1.1.35)
21	Fatty acids	Fatty acid metabolism cluster	3-ketoacyl-CoA thiolase (EC 2.3.1.16)
22	Fatty acids	Fatty acid metabolism cluster	Long-chain-fatty-acid--CoA ligase (EC 6.2.1.3)
23	Fatty acids	Fatty acid metabolism cluster	3-hydroxybutyryl-CoA epimerase (EC 5.1.2.3)

24	Fatty acids	Acyl-CoA thioesterase II	Acyl-CoA thioesterase II (EC 3.1.2.-)
25	Fatty acids	Fatty Acid Biosynthesis FASII	3-hydroxyacyl-[acyl-carrier-protein] dehydratase, FabZ form (EC 4.2.1.59)
26	Fatty acids	Fatty Acid Biosynthesis FASII	3-oxoacyl-[acyl-carrier-protein] synthase, KASI (EC 2.3.1.41)
27	Fatty acids	Fatty Acid Biosynthesis FASII	Acetyl-coenzyme A carboxyl transferase beta chain (EC 6.4.1.2)
28	Fatty acids	Fatty Acid Biosynthesis FASII	Acyl carrier protein
29	Fatty acids	Fatty Acid Biosynthesis FASII	Malonyl CoA-acyl carrier protein transacylase (EC 2.3.1.39)
30	Fatty acids	Fatty Acid Biosynthesis FASII	Acetyl-coenzyme A carboxyl transferase alpha chain (EC 6.4.1.2)
31	Fatty acids	Fatty Acid Biosynthesis FASII	3-hydroxyacyl-[acyl-carrier-protein] dehydratase, FabA form (EC 4.2.1.59)
32	Fatty acids	Fatty Acid Biosynthesis FASII	Biotin carboxylase of acetyl-CoA carboxylase (EC 6.3.4.14)
33	Fatty acids	Fatty Acid Biosynthesis FASII	Biotin carboxyl carrier protein of acetyl-CoA carboxylase
34	Fatty acids	Fatty Acid Biosynthesis FASII	Enoyl-[acyl-carrier-protein] reductase [NADH] (EC 1.3.1.9)

Supplementary Table S4. The genes involved in the biosynthesis of PG, PE, DPG, lipid A and respiratory quinones.

Gene	Function	Acc. numbers
cdsA	phosphatidate cytidylyltransferase	WP_271897010.1
plsC	1-acyl-sn-glycerol-3-phosphate acyltransferase	WP_271895781.1
plsX	phosphate acyltransferase	WP_271893670.1
plsY	glycerol-3-phosphate acyltransferase	WP_271893776.1
pssA	CDP-diacylglycerol—serine O-phosphatidyl transferase	WP_271893933.1
psd	phosphatidylserine decarboxylase	WP_271893483.1
pgsA	CDP-diacylglycerol-glycerol-3-phosphate 3-phosphatidyltransferase	WP_271893933.1
pgpA	phosphatidyl glycerophosphatase	WP_271893599.1
clsA	cardiolipin synthase.	WP_271895714.1
ClsII	eukaryote-like cardiolipin synthase	WP_271893565.1
LpxA	acyl-ACP--UDP-N-acetylglucosamine O-acyltransferase	WP_271896535.1
LpxB	lipid-A-disaccharide synthase	WP_271896537.1

LpxC	UDP-3-O-acyl-N-acetylglucosamine deacetylase	WP_271895068.1
LpxD	UDP-3-O-(3-hydroxymyristoyl)glucosamine N-acyltransferase	WP_271896533.1
LpxH/I	UDP-2,3-diacylglucosamine diphosphatase	WP_271896536.1
ubiA	4-hydroxybenzoate octaprenyltransferase	WP_271894592.1
ubiB	2-polyprenylphenol 6-hydroxylase	WP_271895382.1
ubiE	demethylmenaquinone methyltransferase/2-methoxy-6-polyprenyl-1,4-benzoquinol methylase	WP_271895380.1
ubiG	2-polyprenyl-6-hydroxyphenol methylase/3-demethylubiquinol 3-O-methyltransferase	WP_271897350.1
ubiX	flavin prenyltransferase	WP_271898137.1
pqqC	pyrroloquinoline-quinone synthase	WP_271898008.1

Supplementary Table S5. Several genes in strain IY22 that encode PGP traits

	Category	Subcategory	Role
1	Iron acquisition and metabolism	Siderophores	Ferric hydroxamate ABC transporter (TC 3.A.1.14.3), permease component FhuB
	Iron acquisition and metabolism	Siderophores	N6-hydroxylysine O-acetyltransferase (EC 2.3.1.102), aerobactin biosynthesis protein IucB
	Iron acquisition and metabolism	Siderophores	L-lysine 6-monooxygenase [NADPH] (EC 1.14.13.59), aerobactin biosynthesis protein IucD
	Iron acquisition and metabolism	Siderophores	Iron-chelator utilization protein
	Iron acquisition and metabolism	Siderophores	Ferric hydroxamate ABC transporter (TC 3.A.1.14.3), ATP-binding protein FhuC
	Iron acquisition and metabolism	Siderophores	Ferric hydroxamate ABC transporter (TC 3.A.1.14.3), periplasmic substrate binding protein FhuD
	Iron acquisition and metabolism	Iron acquisition and metabolism - no subcategory	Ferric iron ABC transporter, ATP-binding protein
	Iron acquisition and metabolism	Iron acquisition and metabolism - no subcategory	Ferric iron ABC transporter, iron-binding protein
	Iron acquisition and metabolism	Iron acquisition and metabolism - no subcategory	Ferric iron ABC transporter, permease protein
	Iron acquisition and metabolism	Iron acquisition and metabolism - no subcategory	Hemin transport protein HmuS

	Iron acquisition and metabolism	Iron acquisition and metabolism - no subcategory	Ferrous iron transport peroxidase EfeB
	Iron acquisition and metabolism	Iron acquisition and metabolism - no subcategory	Ferrous iron transport permease EfeU
	Iron acquisition and metabolism	Iron acquisition and metabolism - no subcategory	Ferrous iron transport periplasmic protein EfeO, contains peptidase-M75 domain and (frequently) cupredoxin-like domain
	Iron acquisition and metabolism	Iron acquisition and metabolism - no subcategory	Periplasmic hemin-binding protein
	Iron acquisition and metabolism	Iron acquisition and metabolism - no subcategory	ABC-type hemin transport system, ATPase component
	Iron acquisition and metabolism	Iron acquisition and metabolism - no subcategory	Hemin ABC transporter, permease protein
	Iron acquisition and metabolism	Iron acquisition and metabolism - no subcategory	Hemin transport protein HmuS
2	Nitrogen Metabolism	Nitrogen Metabolism - no subcategory	Nitrogen regulatory protein P-II
	Nitrogen Metabolism	Nitrogen Metabolism - no subcategory	Glutamate-ammonia-ligase adenylyltransferase (EC 2.7.7.42)
	Nitrogen Metabolism	Nitrogen Metabolism - no subcategory	Ammonium transporter
	Nitrogen Metabolism	Nitrogen Metabolism - no subcategory	Glutamine synthetase type II, eukaryotic (EC 6.3.1.2)
	Nitrogen Metabolism	Nitrogen Metabolism - no subcategory	Glutamate synthase [NADPH] large chain (EC 1.4.1.13)
	Nitrogen Metabolism	Nitrogen Metabolism - no subcategory	Glutamine synthetase type I (EC 6.3.1.2)
	Nitrogen Metabolism	Nitrogen Metabolism - no subcategory	[Protein-PII] uridylyltransferase (EC 2.7.7.59)
	Nitrogen Metabolism	Nitrogen Metabolism - no subcategory	Glutamate synthase [NADPH] small chain (EC 1.4.1.13)
3	Virulence, Disease and Defense	Bacteriocins, ribosomally synthesized antibacterial peptides	Conserved uncharacterized protein CreA
	Virulence, Disease and Defense	Resistance to antibiotics and toxic compounds	Cytochrome c heme lyase subunit CcmF
	Virulence, Disease and Defense	Resistance to antibiotics and toxic compounds	Cytochrome c heme lyase subunit CcmH
	Virulence, Disease and Defense	Resistance to antibiotics and toxic compounds	Copper-translocating P-type ATPase (EC 3.6.3.4)
	Virulence, Disease and Defense	Resistance to antibiotics and toxic compounds	Multidrug resistance transporter, Bcr/CflA family

Virulence, Disease and Defense	Resistance to antibiotics and toxic compounds	Cobalt-zinc-cadmium resistance protein
Virulence, Disease and Defense	Resistance to antibiotics and toxic compounds	Heavy metal resistance transcriptional regulator HmrR
Virulence, Disease and Defense	Resistance to antibiotics and toxic compounds	Probable Co/Zn/Cd efflux system membrane fusion protein
Virulence, Disease and Defense	Resistance to antibiotics and toxic compounds	Transcriptional regulator, MerR family
Virulence, Disease and Defense	Resistance to antibiotics and toxic compounds	PF00070 family, FAD-dependent NAD(P)-disulphide oxidoreductase
Virulence, Disease and Defense	Resistance to antibiotics and toxic compounds	Cytoplasmic copper homeostasis protein CutC
Virulence, Disease and Defense	Resistance to antibiotics and toxic compounds	Copper homeostasis protein CutE
Virulence, Disease and Defense	Resistance to antibiotics and toxic compounds	Magnesium and cobalt efflux protein CorC
Virulence, Disease and Defense	Resistance to antibiotics and toxic compounds	DNA gyrase subunit B (EC 5.99.1.3)
Virulence, Disease and Defense	Resistance to antibiotics and toxic compounds	DNA gyrase subunit A (EC 5.99.1.3)
Virulence, Disease and Defense	Resistance to antibiotics and toxic compounds	Metal-dependent hydrolases of the beta-lactamase superfamily I
Virulence, Disease and Defense	Resistance to antibiotics and toxic compounds	Multi antimicrobial extrusion protein (Na(+)/drug antiporter), MATE family of MDR efflux pumps
Virulence, Disease and Defense	Resistance to antibiotics and toxic compounds	Multidrug and toxin extrusion (MATE) family efflux pump YdhE/NorM, homolog
Virulence, Disease and Defense	Resistance to antibiotics and toxic compounds	Macrolide-specific efflux protein MacA
Virulence, Disease and Defense	Resistance to antibiotics and toxic compounds	Chromate transport protein ChrA
Virulence, Disease and Defense	Invasion and intracellular resistance	SSU ribosomal protein S7p (S5e)
Virulence, Disease and Defense	Invasion and intracellular resistance	Translation elongation factor G
Virulence, Disease and Defense	Invasion and intracellular resistance	Translation elongation factor Tu
Virulence, Disease and Defense	Invasion and intracellular resistance	SSU ribosomal protein S12p (S23e)
Virulence, Disease and Defense	Invasion and intracellular resistance	DNA-directed RNA polymerase beta' subunit (EC 2.7.7.6)
Virulence, Disease and Defense	Invasion and intracellular resistance	DNA-directed RNA polymerase beta subunit (EC 2.7.7.6)
Virulence, Disease and Defense	Invasion and intracellular resistance	Quinolinate synthetase (EC 2.5.1.72)

	Virulence, Disease and Defense	Invasion and intracellular resistance	Quinolinate phosphoribosyltransferase [decarboxylating] (EC 2.4.2.19)
	Virulence, Disease and Defense	Invasion and intracellular resistance	L-aspartate oxidase (EC 1.4.3.16)
	Virulence, Disease and Defense	Invasion and intracellular resistance	LSU ribosomal protein L35p
	Virulence, Disease and Defense	Invasion and intracellular resistance	Translation initiation factor 3
	Virulence, Disease and Defense	Invasion and intracellular resistance	LSU ribosomal protein L20p
4	Membrane Transport	Protein secretion system, Type II	Type IV prepilin peptidase TadV/CpaA
	Membrane Transport	Protein secretion system, Type II	Flp pilus assembly protein TadB
	Membrane Transport	Protein secretion system, Type II	Flp pilus assembly protein RcpC/CpaB
	Membrane Transport	Protein secretion system, Type II	Type II/IV secretion system protein TadC, associated with Flp pilus assembly
	Membrane Transport	Protein secretion system, Type II	Type II/IV secretion system ATPase TadZ/CpaE, associated with Flp pilus assembly
	Membrane Transport	Protein secretion system, Type II	Type II/IV secretion system ATP hydrolase TadA/VirB11/CpaF, TadA subfamily
	Membrane Transport	Protein secretion system, Type II	Flp pilus assembly protein CpaD
	Membrane Transport	Protein secretion system, Type II	Similar to secretin RcpA/CpaC, associated with Flp pilus assembly
	Membrane Transport	Protein secretion system, Type II	Type II/IV secretion system secretin RcpA/CpaC, associated with Flp pilus assembly
	Membrane Transport	Protein secretion system, Type II	Flp pilus assembly protein, pilin Flp
	Membrane Transport	Protein secretion system, Type II	Flp pilus assembly protein TadD, contains TPR repeat
	Membrane Transport	ABC transporters	Oligopeptide transport ATP-binding protein OppF (TC 3.A.1.5.1)
	Membrane Transport	Protein secretion system, Type VII (Chaperone/Usher pathway, CU)	Sigma-fimbriae usher protein
	Membrane Transport	Protein secretion system, Type VII (Chaperone/Usher pathway, CU)	Sigma-fimbriae chaperone protein
	Membrane Transport	Protein translocation across cytoplasmic membrane	Twin-arginine translocation protein TatC
	Membrane Transport	Protein translocation across cytoplasmic membrane	Twin-arginine translocation protein TatA

Membrane Transport	Protein translocation across cytoplasmic membrane	Twin-arginine translocation protein TatB
Membrane Transport	Cation transporters	Magnesium and cobalt transport protein CorA
Membrane Transport	Cation transporters	Magnesium and cobalt efflux protein CorC
Membrane Transport	Cation transporters	HoxN/HupN/NixA family nickel/cobalt transporter
Membrane Transport	Cation transporters	Nickel responsive regulator NikR
Membrane Transport	Cation transporters	Predicted cobalt transporter CbtA
Membrane Transport	Cation transporters	Copper-translocating P-type ATPase (EC 3.6.3.4)
Membrane Transport	Cation transporters	Copper binding protein, plastocyanin/azurin family
Membrane Transport	Uni- Sym- and Antiporters	Na+/H+ antiporter NhaA type
Membrane Transport	Uni- Sym- and Antiporters	Sodium-dependent phosphate transporter
Membrane Transport	Membrane Transport - no subcategory	TolA protein
Membrane Transport	Membrane Transport - no subcategory	Protein-L-isoaspartate O-methyltransferase (EC 2.1.1.77)
Membrane Transport	Membrane Transport - no subcategory	Tol biopolymer transport system, TolR protein
Membrane Transport	Membrane Transport - no subcategory	Iron-chelator utilization protein
Membrane Transport	TRAP transporters	TRAP transporter solute receptor, unknown substrate 6
Membrane Transport	TRAP transporters	TRAP dicarboxylate transporter, DctM subunit, unknown substrate 6
Membrane Transport	TRAP transporters	TRAP dicarboxylate transporter, DctQ subunit, unknown substrate 6
Membrane Transport	TRAP transporters	TRAP-type C4-dicarboxylate transport system, small permease component
Membrane Transport	TRAP transporters	TRAP-type C4-dicarboxylate transport system, large permease component
Membrane Transport	TRAP transporters	TRAP-type C4-dicarboxylate transport system, periplasmic component
Membrane Transport	Protein and nucleoprotein secretion system, Type IV	Lipoprotein of type IV secretion complex that spans outer membrane and periplasm, VirB7
Membrane Transport	Protein and nucleoprotein secretion system, Type IV	ATPase required for both assembly of type IV secretion complex and secretion of T-DNA complex, VirB11

	Membrane Transport	Protein and nucleoprotein secretion system, Type IV	T-DNA border endonuclease VirD2, RP4 TraG-like relaxase
	Membrane Transport	Protein and nucleoprotein secretion system, Type IV	Minor pilin of type IV secretion complex, VirB5
	Membrane Transport	Protein and nucleoprotein secretion system, Type IV	ATPase required for both assembly of type IV secretion complex and secretion of T-DNA complex, VirB4
	Membrane Transport	Protein and nucleoprotein secretion system, Type IV	Inner membrane protein forms channel for type IV secretion of T-DNA complex, VirB8
	Membrane Transport	Protein and nucleoprotein secretion system, Type IV	Inner membrane protein forms channel for type IV secretion of T-DNA complex, VirB3
	Membrane Transport	Protein and nucleoprotein secretion system, Type IV	Coupling protein VirD4, ATPase required for T-DNA transfer
	Membrane Transport	Protein and nucleoprotein secretion system, Type IV	Inner membrane protein of type IV secretion of T-DNA complex, TonB-like, VirB10
	Membrane Transport	Protein and nucleoprotein secretion system, Type IV	Outer membrane and periplasm component of type IV secretion of T-DNA complex, has secretin-like domain, VirB9
	Membrane Transport	Protein and nucleoprotein secretion system, Type IV	Major pilus subunit of type IV secretion complex, VirB2
	Membrane Transport	Protein and nucleoprotein secretion system, Type IV	Inner membrane protein of type IV secretion of T-DNA complex, VirB6
	Membrane Transport	Protein and nucleoprotein secretion system, Type IV	ATPase required for both assembly of type IV secretion complex and secretion of T-DNA complex, VirB11
	Membrane Transport	Protein and nucleoprotein secretion system, Type IV	Single-stranded DNA-binding protein
	Membrane Transport	Protein and nucleoprotein secretion system, Type IV	ATPase required for both assembly of type IV secretion complex and secretion of T-DNA complex, VirB4
	Membrane Transport	Protein and nucleoprotein secretion system, Type IV	Inner membrane protein forms channel for type IV secretion of T-DNA complex, VirB8
	Membrane Transport	Protein and nucleoprotein secretion system, Type IV	DNA topoisomerase I (EC 5.99.1.2)
	Membrane Transport	Protein and nucleoprotein secretion system, Type IV	Coupling protein VirD4, ATPase required for T-DNA transfer
5	Stress Response	Osmotic stress	Aquaporin Z
	Stress Response	Osmotic stress	Cyclic beta-1,2-glucan synthase (EC 2.4.1.-)
	Stress Response	Osmotic stress	2)glucan export ATP-binding/permease protein NdvA (EC 3.6.3.42)Beta-(1-->2)glucan export ATP-binding/permease protein NdvA (EC 3.6.3.42)
	Stress Response	Osmotic stress	OpgC protein
	Stress Response	Osmotic stress	Cyclic beta-1,2-glucan modification transmembrane protein

Stress Response	Osmotic stress	Sarcosine oxidase gamma subunit (EC 1.5.3.1)
Stress Response	Osmotic stress	Sarcosine oxidase alpha subunit (EC 1.5.3.1)
Stress Response	Osmotic stress	Sarcosine oxidase beta subunit (EC 1.5.3.1)
Stress Response	Osmotic stress	Choline-sulfatase (EC 3.1.6.6)
Stress Response	Osmotic stress	Betaine aldehyde dehydrogenase (EC 1.2.1.8)
Stress Response	Osmotic stress	HTH-type transcriptional regulator BetI
Stress Response	Osmotic stress	GbcA Glycine betaine demethylase subunit A
Stress Response	Osmotic stress	Sarcosine oxidase delta subunit (EC 1.5.3.1)
Stress Response	Osmotic stress	L-proline glycine betaine binding ABC transporter protein ProX (TC 3.A.1.12.1)
Stress Response	Osmotic stress	Choline dehydrogenase (EC 1.1.99.1)
Stress Response	Oxidative stress	Superoxide dismutase [Fe] (EC 1.15.1.1)
Stress Response	Oxidative stress	Superoxide dismutase [Mn] (EC 1.15.1.1)
Stress Response	Oxidative stress	Organic hydroperoxide resistance transcriptional regulator
Stress Response	Oxidative stress	Iron-responsive regulator Irr
Stress Response	Oxidative stress	Alkyl hydroperoxide reductase subunit C-like protein
Stress Response	Oxidative stress	Superoxide dismutase [Fe] (EC 1.15.1.1)
Stress Response	Oxidative stress	Phytochrome, two-component sensor histidine kinase (EC 2.7.3.-)
Stress Response	Oxidative stress	Organic hydroperoxide resistance protein
Stress Response	Oxidative stress	Gamma-glutamyltranspeptidase (EC 2.3.2.2)
Stress Response	Oxidative stress	Glutathione synthetase (EC 6.3.2.3)
Stress Response	Oxidative stress	Glutamate--cysteine ligase (EC 6.3.2.2)
Stress Response	Oxidative stress	Glutathione S-transferase, zeta (EC 2.5.1.18)

Stress Response	Oxidative stress	Glutathione S-transferase (EC 2.5.1.18)
Stress Response	Oxidative stress	Uncharacterized glutathione S-transferase-like protein
Stress Response	Oxidative stress	Lactoylglutathione lyase (EC 4.4.1.5)
Stress Response	Oxidative stress	Glutathione S-transferase, unnamed subgroup (EC 2.5.1.18)
Stress Response	Oxidative stress	Glutathione S-transferase, omega (EC 2.5.1.18)
Stress Response	Oxidative stress	Glutathione S-transferase family protein
Stress Response	Oxidative stress	Hydroxyacylglutathione hydrolase (EC 3.1.2.6)
Stress Response	Oxidative stress	Glutathione reductase (EC 1.8.1.7)
Stress Response	Oxidative stress	Uncharacterized monothiol glutaredoxin ycf64-like
Stress Response	Oxidative stress	Glutaredoxin 3 (Grx2)
Stress Response	Oxidative stress	Uncharacterized monothiol glutaredoxin ycf64-like
Stress Response	Oxidative stress	Glutaredoxin 3 (Grx2)
Stress Response	Detoxification	Sulfate and thiosulfate import ATP-binding protein CysA (EC 3.6.3.25)
Stress Response	Detoxification	S-formylglutathione hydrolase (EC 3.1.2.12)
Stress Response	Detoxification	S-(hydroxymethyl)glutathione dehydrogenase (EC 1.1.1.284)
Stress Response	Stress Response - no subcategory	Ornithine aminotransferase (EC 2.6.1.13)
Stress Response	Stress Response - no subcategory	NG,NG-dimethylarginine dimethylaminohydrolase 1 (EC 3.5.3.18)
Stress Response	Stress Response - no subcategory	Putative inner membrane protein YjeT (clustered with HflC)
Stress Response	Stress Response - no subcategory	HflC protein
Stress Response	Stress Response - no subcategory	RNA-binding protein Hfq
Stress Response	Stress Response - no subcategory	HflK protein
Stress Response	Periplasmic Stress	Intramembrane protease RasP/YluC, implicated in cell division based on FtsL cleavage

	Stress Response	Periplasmic Stress	HtrA protease/chaperone protein
6	Sulfur Metabolism	Inorganic sulfur assimilation	Conserved hypothetical protein probably involved in sulfate reduction
	Sulfur Metabolism	Inorganic sulfur assimilation	Adenylylsulfate kinase (EC 2.7.1.25)
	Sulfur Metabolism	Inorganic sulfur assimilation	Sulfate and thiosulfate binding protein CysP
	Sulfur Metabolism	Inorganic sulfur assimilation	3'(2'),5'-bisphosphate nucleotidase (EC 3.1.3.7)
	Sulfur Metabolism	Inorganic sulfur assimilation	Sulfate transport system permease protein CysW
	Sulfur Metabolism	Inorganic sulfur assimilation	Oxidoreductase probably involved in sulfite reduction
	Sulfur Metabolism	Inorganic sulfur assimilation	Sulfate transport system permease protein CysT
	Sulfur Metabolism	Inorganic sulfur assimilation	Phosphoadenylyl-sulfate reductase [thioredoxin] (EC 1.8.4.8)
	Sulfur Metabolism	Inorganic sulfur assimilation	Sulfate adenylyltransferase subunit 1 (EC 2.7.7.4)
	Sulfur Metabolism	Inorganic sulfur assimilation	Sulfate adenylyltransferase subunit 2 (EC 2.7.7.4)
	Sulfur Metabolism	Inorganic sulfur assimilation	Sulfite reductase [NADPH] hemoprotein beta-component (EC 1.8.1.2)
	Sulfur Metabolism	Inorganic sulfur assimilation	Sulfate and thiosulfate import ATP-binding protein CysA (EC 3.6.3.25)
	Sulfur Metabolism	Inorganic sulfur assimilation	Ferredoxin
	Sulfur Metabolism	Sulfur Metabolism - no subcategory	Thiol peroxidase, Bcp-type (EC 1.11.1.15)
	Sulfur Metabolism	Sulfur Metabolism - no subcategory	Alkyl hydroperoxide reductase subunit C-like protein
	Sulfur Metabolism	Sulfur Metabolism - no subcategory	Thioredoxin reductase (EC 1.8.1.9)
	Sulfur Metabolism	Sulfur Metabolism - no subcategory	Arylsulfatase (EC 3.1.6.1)
	Sulfur Metabolism	Organic sulfur assimilation	Alkanesulfonates ABC transporter ATP-binding protein
